# Effect of Extraction Methods on Aroma Profile, Antioxidant Activity and Sensory Acceptability of Specialty Coffee Brews

**DOI:** 10.3390/foods12224125

**Published:** 2023-11-14

**Authors:** Barbora Lapčíková, Lubomír Lapčík, Petr Barták, Tomáš Valenta, Kateřina Dokládalová

**Affiliations:** 1 Department of Physical Chemistry, Faculty of Science, Palacky University Olomouc, 17. Listopadu 12, 771 46 Olomouc, Czech Republic or lapcikova@utb.cz (B.L.); petr.bartak@upol.cz (P.B.); 2 Department of Food Technology, Faculty of Technology, Tomas Bata University in Zlín, nám. T. G. Masaryka 5555, 760 01 Zlín, Czech Republic; tvalenta@utb.cz (T.V.); dokladalova.katerina22@gmail.com (K.D.)

**Keywords:** specialty coffee, roasting degree, coffee brew, antioxidant activity, aroma profile, sensory analysis

## Abstract

Specialty coffees from various geographical origins were processed using different extraction methods. Four extraction techniques were employed: cold brew (CB), espresso (ES), French press (FR), and aeropress (AE). The potential health benefits of coffee brews were linked to their antioxidant activity, as determined by the DPPH assay, and total polyphenol content (TPC) measured through the Folin–Ciocalteu reducing-capacity assay. The Columbia (C) espresso coffee type (omni-roasting) exhibited the highest antioxidant activity (86.31 ± 0.70) μmol/100 mL, with a TPC value of (44.41 ± 0.35) mg GAE/g. Quantitative analyses of caffeine and chlorogenic acid were conducted using high-performance liquid chromatography (HPLC). The evaluation of coffee aroma profiles involved the application of headspace solid-phase microextraction/gas chromatography–mass spectrometry (HS-SPME/GC-MS) and was complemented by sensory analysis following the Specialty Coffee Association (SCA) standard protocol. The predominant volatile compounds found in all samples included furans, phenols, pyrazines, and terpenes. The EY espresso type (medium dark roasting) had the highest levels of most coffee volatiles. The C cold brew type (omni-roasting) was rated as the preferred coffee in terms of its sensory characteristics and flavour. In summary, ES and CB were found to be more effective extraction methods for the parameters assessed.

## 1. Introduction

*Coffea arabica* L. is a globally cultivated coffee species used in the production of various coffee beverages. Espresso is a popular type of coffee brew prepared by percolating hot water under pressure through a cake of roasted ground coffee to extract the flavour. The water pressure and temperature significantly influence the brew’s properties and quality. However, certain variables during preparation, such as the coffee particle size and powder compression, may be beyond control but can have a significant impact on the final brew’s properties [1].

Cold brew extraction has emerged as a recent trend in the coffee industry. This method involves extracting medium-roasted coffee (at a concentration of 50–100 g/L) with water at a temperature lower than body temperature. Traditional cold brew coffee takes an extended time to extract (up to 24 h), which reduces production efficiency. However, recent studies have shown that most compounds can be extracted within just a few hours. Increasing the temperature and water flow turbulence (e.g., using ultrasonication) can further accelerate the extraction process [2,3,4]. The aroma compounds in cold brews significantly differ from those in hot brews [2,5] and offer a sensory profile preferred over that of static cold coffee beverages [3].

The French press method is an immersion-style brewing process where coffee is steeped in hot water and then separated by pressing down on a metal mesh filter. This method allows the coffee grounds to steep directly into hot water, extracting a range of flavours and oils that would otherwise be trapped by paper filters in a traditional drip brew machine [6]. The aeropress method, similar to espresso, employs manual pressure to extract flavour. It uses finely ground beans and has a short brewing time. Water pressure and temperature significantly influence the brew’s properties and quality. Both the aeropress and French press methods can produce coffee with a well-liked flavour and relatively high extraction efficiency [7,8].

HS-SPME/GC-MS (headspace solid-phase microextraction/gas chromatography–mass spectrometry) is an analytical tool that is widely used to characterize the volatile compounds of coffee beverages. This technique can provide valuable insights into the effects of different extraction methods on the coffee’s volatile compounds profile. It can also aid in understanding how diverse factors influence the coffee aroma. A wide range of volatile compounds, including aldehydes, ketones, acids, esters, alcohols, and others, contribute to the complex aroma of coffee [9]. Current studies have been focused on the characterization of volatile compounds from green berries to roasted coffee and coffee brews, as well as on the influence of geographical origin on the overall coffee aroma profile, applying the HS-SPME/GC-MS technique [10,11,12].

Coffee is considered a functional food due to its inherent antioxidant properties. It contains bioactive compounds such as caffeine, chlorogenic acid, tannic acid, nicotinic acid, catechins, anthocyanins, ferulic acids, and caffeic acid. Coffee’s antioxidant activity is primarily attributed to the presence of polyphenols, flavonoids, and other bioactive compounds, representing a key health benefit of coffee consumption [13,14,15]. Caffeine and chlorogenic acid isomers are key contributors to coffee’s final flavour, including the astringency, bitterness, and metallic taste. These compounds are also linked to the health-promoting aspects of coffee consumption. The physicochemical properties and sensory attributes of coffee beverages depend on the processing of the coffee beans, roasting, and brewing [9].

During the processing of coffee beans and brew preparation, essential quality characterization factors like the colour, aroma, and flavour are developed [16,17]. Different roasting (drying) methods can result in specific volatile compound profiles due to varying dehydration behaviours within the coffee beans [18]. Roasted coffee is characterized by its roast degree, which influences the external bean colour (ranging from light to dark brown), flavour, dry matter loss, and changes in the coffee composition [19]. Roasting involves complex reactions that generate high-molecular-weight compounds called melanoidins through carbohydrate caramelization or Maillard reactions between sugars and amino acids, which also contribute to coffee’s aroma by forming heterocyclic molecules. Roasted coffee and brewed coffee share most chemical compounds, regardless of the brewing method, with slight changes in aroma due to shifts in the concentration of aroma substances during brewing [20,21]. The overextraction of coffee results in an excess of bitter flavour, influenced by the grinding degree [22]. Optimal grinding and brewing combinations can produce a consistent coffee brew with a complex flavour, intense aroma, and balanced acidity and sweetness [23,24].

The objective of this study was to evaluate the impact of coffee brew preparation on the aroma profile of specialty coffee beans representing different variants from Africa, Central America, and South America. Four different extraction methods (cold brew, espresso, French press, and aeropress) were employed based on their various time/temperature-dependent extraction kinetics. The HS-SPME/GC-MS method was used to identify the volatile components present in the coffee brews. Antioxidant activity’s relation to bioactive compounds was assessed, and the content of all polyphenols, caffeine, and chlorogenic acid was evaluated in relation to the coffee extraction method. Sensory attributes were rated following the procedure of the Specialty Coffee Association (SCA) (Santa Ana, CA, USA) and evaluated in correlation with the coffee brew types.

## 2. Materials and Methods

### 2.1. Coffee Beans

Five specialty varieties of plantation green coffee beans (*Coffea arabica* L.) were carefully selected and acquired in collaboration with the coffee roaster company, Pražírna kávy KIKAFE s.r.o., located in Olomouc, Czechia. These coffee bean varieties are detailed in Table 1. The samples were stored in airtight polyethylene food packages and kept in a dark environment at the ambient laboratory temperature of (24 ± 1) °C.

### 2.2. Chemicals and Reagents

Reagents and chemicals of analytical grade were used in this study. DPPH (1,1-diphenyl-2-picrylhydrazyl) with 95% purity was provided by Alfa Aesar (Ward Hill, MA, USA). Trolox, with 97% purity, methanol at 99.9%, acetonitrile at 99.9%, 85% phosphoric acid (H_3_PO_4_), and 99% gallic acid were sourced from Sigma-Aldrich (Burlington, MA, USA). Folin–Ciocalteu’s phenol reagent at a concentration of 2 M and crystalline chlorogenic acid with 95% purity were obtained from Merck KGaA (Darmstadt, Germany). The caffeine standard (1,3,7-trimethylpurine-2,6-dione) with 100% purity and sodium carbonate with 99.7% purity were supplied by Penta Ltd. (Prague, Czech Republic) and IPL (Otrokovice, Czech Republic), respectively.

### 2.3. Roasting and Grinding of Coffee Beans

The coffee beans were roasted at the coffee-roaster company using the Probat Probatone 5 roaster (Probat, AG, Ingolstadt, Germany). Two roasting methods, light and medium, were employed, following the specific parameters required for the preparation of espresso and filter roasts. Light roasting (omni-roast) was applied to the Columbia (C) coffee type, as a result of the carbonic maceration processing used for this coffee (as indicated in Table 2). Omni-roast is suitable for both espresso and filtered coffees. The roasting process was initiated at a temperature of 210 °C by introducing the coffee beans into the roaster. The temperature was then allowed to decrease to 125 °C. The colour of the coffee beans was monitored at 155 °C using a propane gas inlet. At temperatures around 170 °C, the coffee beans caramelized during the roasting process, which was followed by the initial bursting of the beans. After reaching a temperature of 214–215 °C, the coffee beans were cooled by air in a drum. Variations in the roasting of coffee beans were based on differences in the initial bean weight, roasting temperature, and time.

Following roasting, the coffee beans were left to rest for four days in special paper valve bags before being ground using the Mahlkönig EK43 coffee grinder (ADLER Europe Group, Warsaw, Poland), with the grinder scale (0–16). The degree of coarseness was selected based on the ideal granularity suitable for the various types of coffee brewing. The finest grinding was used for espresso, medium grinding (between drip and espresso grinds) for aeropress, and the most intense (coarse) grinding for cold brew and French press. The grinding, roasting, and brewing combinations (Table 2) were carefully selected to prepare consistent coffee brew profiles, each typical for the respective coffee type.

### 2.4. Particle Size Determination by Sieve Analysis

Ground coffee samples underwent sieving using the Vibratory Sieve Shaker Analysette 3 (Fritsch, GmbH—Milling and Sizing, Idar-Oberstein, Germany). The sieves with various mesh diameters were arranged in the following sequence: 900, 710, 560, 450, 320, 220, 160, 125, and 45 μm. The sieving process lasted 5 min, and the masses of individual fractions retained on the sieves were determined. The analysis results are presented as the mass ratio dependency of particle fractions on particle radius. The mean particle diameter, *d_i_*, retained between the sieves of linear mesh sizes *b_i_* and *b_i−_*_1_, respectively, was calculated as the arithmetic mean of the mesh sizes [25]:

(1) $$d_{i}=\left(b_{i-1}+b_{i}\right)/2$$

The weight average (mean) particle radius $\bar{r_{w}}$ was calculated by the following equation:

(2) $$\bar{r_{w}}=\frac{\sum \left(r_{i}\cdot m_{i}\right)}{\sum m_{i}}$$

where *r_i_* is the average mesh radius (calculated from Equation (1)), and *m_i_* is the weight of the particles fraction with radius *r_i_* on the mesh. The resulting grind sizes are detailed in Table 2.

### 2.5. Coffee Brews Preparation

Coffee brews (CBs) were prepared by various extraction methods. In all experiments, tap drinking water with a conductivity of 25 mS/m was used. The quality of the drinking water complied with the EC COUNCIL DIRECTIVE 98/83/EC of 3 November 1998 on the quality of water intended for human consumption [26].

For the cold brew (CB), we combined 25 g of ground coffee with laboratory-temperature water (24 ± 1) °C to yield 250 mL of brew. The maceration process continued for 12 h.

In the case of espresso (ES), we utilized the KRUPS Calvi XP 3440 lever coffee maker (KRUPS, Solingen, Germany). Seven grams of ground coffee were introduced into the machine to produce a 25 mL coffee drink. The pump pressure was set at 15 bar, the water extraction temperature was maintained at 90 °C, and the extraction time was 30 s.

To prepare the French press (FR), the IKEA French press coffee machine (IKEA, Prague, Czech Republic) was employed. Using 15 g of ground coffee for every 250 mL of the brew, the sample was stirred for 30 s to ensure even distribution of the grounds with water. After stirring, the coffee steeped for 4 min before the plunger was pressed down. The water extraction temperature was 93 °C, and the extraction time was 3.5 min.

For the aeropress (AE), the AeroPress Aerobie model (AeroPress, Inc., Palo Alto, CA, USA) with original microfilters was utilized, applying 18 g of ground coffee per 250 mL of the beverage. To bloom the coffee, the water was poured twice over the coffee bed, from the centre to the sides, and kept for 30 s to allow the carbon dioxide to be released. The water extraction temperature was 93 °C, and the extraction process lasted 1.25 min.

Following preparation, the coffee samples were filtered using syringe microfilters with a pore size of 0.20 µm (Sigma-Aldrich, Burlington, MA, USA). To standardize the different coffee-to-water ratios, the analysis data were calculated using an appropriate dilution factor for the extraction methods applied. The obtained results were expressed on the basis of coffee weight and further presented per 100 mL of coffee cup. Extraction efficiency in terms of how much coffee soluble substances were extracted into the water was dependent on the factors like coffee grind size, brewing time, and water temperature, as specified for the methods applied. For the analyses, a set of coffee brew extracts was prepared. Each sample was analysed in triplicate; for each type of analysis, one extract from the set of the coffee brew was used.

### 2.6. DPPH Radical Scavenging Activity Measurement

The method was based on the reaction of a test coffee sample with the stable radical DPPH (1,1-diphenyl-2-(2,4,6-trinitrophenyl)hydrazyl). During this reaction, the radical was reduced to its DPPH-H form. The spectrophotometric monitoring of the reaction between scavengers and the DPPH radical allowed us to determine the dynamic relationship between absorbance and reaction time [27,28].

To perform the test, 0.1 mL of filtered coffee was added to 5 mL of a DPPH methanol solution in a test tube and vigorously shaken. The test tubes were then incubated in the dark at room temperature (24 ± 1) °C, and samples were measured at specific time intervals (0, 15, 30, 45, and 60 min). Standardization was performed using Trolox, which was dissolved in methanol to reach a concentration of 200 mg/L. The calibration range was prepared in the following concentrations: 20, 40, 80, 100, 120, and 160 mg/L. A control sample was prepared without active substances, and methanol was used for baseline correction. Changes in the absorbance of samples were measured at 515 nm by a UV-Vis spectrophotometer (CECIL CE 1021, Cecil Instruments Ltd., Peterborough, Cambridgeshire, UK). Radical-scavenging activity was expressed as a percentage of inhibition using the following formula:

(3) $$Inactivation=\frac{A_{0}-A_{c}}{A_{0}}\times 100\%$$

where *A*_0_ is the absorbance at 515 nm without coffee extract, and *A_c_* is the absorbance with the coffee extract [29,30].

### 2.7. Total Polyphenols Content (TPC) Assay by Folin–Ciocalteu Method

To determine the total content of phenolic substances in coffee samples, the Folin–Ciocalteu reducing-capacity method was employed. This method involves the reduction of the Folin–Ciocalteu reagent in the presence of polyphenols, resulting in the formation of a molybdenum–tungsten blue complex, which was measured spectrophotometrically at 764 nm [31,32].

For the analysis, the samples were prepared by mixing 100 μL of diluted coffee brews with 300 μL of Folin–Ciocalteu reagent. After a 3-min reaction, the solution was basified by adding 0.5 mL of 14 wt.% sodium carbonate and completed with 4 mL of distilled water. The samples were left to react for 60 min in a dark environment. Subsequently, the absorbance was measured using a UV-Vis spectrophotometer (CECIL CE 1021, Cecil Instruments Ltd., Peterborough, Cambridgeshire, UK) at a wavelength of 765 nm, with a blank sample (without coffee extract) as the reference.

The total content of polyphenolic compounds in coffee samples was calculated as the gallic acid equivalent (mg of GAE/g of ground coffee). Calibration solutions within the range of 5–50 mg/mL of gallic acid were prepared to generate the calibration curve (y = 0.01x + 0.0115 with a correlation coefficient of 0.9988). Using this calibration curve, the absorbance values were converted to determine the total phenolic content (TPC) of the coffee samples [33].

### 2.8. Caffeine and Chlorogenic Acid Contents by HPLC Analysis

Standard stock solutions with defined concentrations were prepared, including a 4.5 mg/mL caffeine standard and a 1 mg/mL chlorogenic acid standard. Subsequently, calibration sets were established for both standards. The caffeine calibration solutions were prepared from the stock solution within the calibration range of 0.225–1.125 mg/mL, while the chlorogenic acid calibration set ranged from 0.05 mg/mL to 0.25 mg/mL.

High-performance liquid chromatography (HPLC) was conducted using the method developed by Parenti et al. [1], with some modifications based on the latest knowledge from Klikarová et al. [9]. A Dionex Ultimate 3000 chromatograph (Thermo Fisher Scientific, Waltham, MA, USA) was utilized. Chromatographic separation was achieved using a reversed-phase Kinetex EVO C-18 column (Phenomenex, Torrance, CA, USA) with dimensions of 150 × 4.6 mm and a particle size of 2.6 μm. Each sample consisted of 10 μL, and the coffee extracts were filtered through 0.20 μm filters, placed in 3 mL vials, and analysed in triplicate. The HPLC run-time was 54 min, with a flow rate ranging from 0.5 mL/min to 0.8 mL/min. The column temperature was maintained at 25 °C. An isocratic elution was performed using a constant mobile phase, which consisted of a 0.05% phosphoric acid aqueous solution. The detection process utilized UV-Vis at a wavelength of 210 nm. Caffeine and chlorogenic acid were identified based on their retention times using commercial standards. Concentrations of caffeine and chlorogenic acid in the analysed samples were calculated from HPLC peak areas using linear regression equations of the relevant calibration curves: for caffeine, y = 1217.2x − 2.5241 with a correlation coefficient of 0.9994, and for chlorogenic acid, y = 323.3183x + 1.2710 with a correlation coefficient of 0.9990. The concentrations were expressed in mg/100 mL of coffee extracts [34].

The parameters of analysis validation included identification of detection and quantification limits. Limit of detection (LOD) was determined by the following equation:

(4) LOD=3.3×(σ/S)

where *σ* means the standard deviation of the response, and *S* the mean slope of the calibration curve.

Limit of quantification (LOQ) was determined using Equation (5):

(5) LOQ=10×(σ/S)

where *σ* represents the standard deviation of the response, and *S* represents the mean slope of the calibration curve [35].

### 2.9. Aroma Profile of Coffee Brews by HS-SPME/GC-MS

Volatile compounds that characterize the aroma profile of coffee brews were detected using gas chromatography–mass spectrometry (GC-MS) with solid-phase microextraction operated in head-space mode (HS-SPME). In this process, 5 mL of coffee brew was poured into a septum-sealed head-space vial (40 mL) with a magnetic stirring bar (500 rpm) and extracted using a divinylbenzene/carboxen/polydimethylsiloxane extraction fibre. This extraction was carried out with a manual sampling holder (Supelco, Bellefonte, PA, USA) for 80 min at 50 °C.

All analyses were conducted on the HP 6890 Series GC system with the Agilent 5973 N Mass Selective Detector (Agilent, Palo Alto, CA, USA). It was equipped with an HP-5 ms capillary column (30 m × 0.25 mm × 0.25 µm) operating with a temperature program starting at 40 °C for 1 min and then increasing to 200 °C at a rate of 3 °C/min. Finally, it reached 300 °C and was held for 5 min at a rate of 10 °C/min. Helium (99.9995%, SIAD, Bergamo, Italy) was used as the carrier gas at a flow rate of 0.9 mL/min, and desorption was performed in splitless mode at 270 °C for 5 min with a purge time of 0.3 min. Mass scans were recorded within the range of 29–520 *m*/*z* using electron impact ionization at 70 eV. The temperatures of the transfer line, ion source, and quadrupole were set at 280 °C, 230 °C, and 150 °C, respectively. The method repeatability expressed as the relative standard deviation of peaks areas from five repeated measurements ranged from 7 to 19% for the main components of the samples.

### 2.10. Sensory Analysis of Coffee Brews

Sensory analysis was performed using the standard protocol of the Specialty Coffee Association, which is the qualified process for the evaluation of fine coffees [36]. Brewed coffee samples were tested at standard temperatures specified for individual sensory attributes, as described below [37,38]. Seven members of the sensory panel, both men and women at the age of 20–40 years, participated in sensory analysis. The quality attributes were evaluated by a sequence of rating where each attribute was based on the flavour perception related to decreasing coffee temperature. The evaluation procedure was as follows: step 1—aroma/fragrance (3–5 min after coffee preparation); step 2—flavour, aftertaste, acidity, body, and balance (in temperature range 60–70 °C); step 3—sweetness, uniformity, cleanliness, and overall (at temperature ≤ 37.8 °C). The quality scale was presented in four categories: good (6–6.75), very good (7–7.75), excellent (8–8.75), and outstanding (9–9.75). Final scoring was calculated by summing the individual scores given for each attribute and classifying them into corresponding categories for specialty coffee of outstanding quality (90–100), excellent quality (85–89.99), and very good quality (80–84.99). The coffees below speciality quality were characterized by a final score of <80.

### 2.11. Statistical Analysis

Data from physicochemical analyses were subjected to statistical evaluation using a one-way analysis of variance (ANOVA) method. Differences in the mean values among the statistical groups were tested at a significance level of α ≤ 0.05. The Tukey test was applied for multiple comparisons of the mean responses among treatment groups to assess statistical significance, i.e., to evaluate if the differences were greater than what would be expected by chance; different superscript letters are used to indicate statistically significant differences between the values determined. Data testing was performed using the statistical software SigmaStat version 2.03 (Systat Software, Inc., San Jose, CA, USA). The results were expressed as arithmetic mean ± standard deviation.

The sensory analysis was assessed using the Pearson chi-square test at a significance level of α ≤ 0.05. The Pearson test was employed to verify the independence of the tested variables, which included coffee type and preparation method, with respect to the total scores of the sensory attributes. The variable frequencies provided by the panellists’ preferences were compared to the expected frequencies based on the validity of the null hypothesis H, which assumed that there were no significant differences between coffee samples in the observed sensory attributes (preferences). The alternative hypothesis A suggested that a dependent relationship existed between the variables. Using the Pearson test, the validity of hypothesis H was verified through the test criterion X with degrees of freedom of (r − 1) × (c − 1).

## 3. Results and Discussion

### 3.1. Antioxidant Activity of Coffee Brews

**Figure 1:**
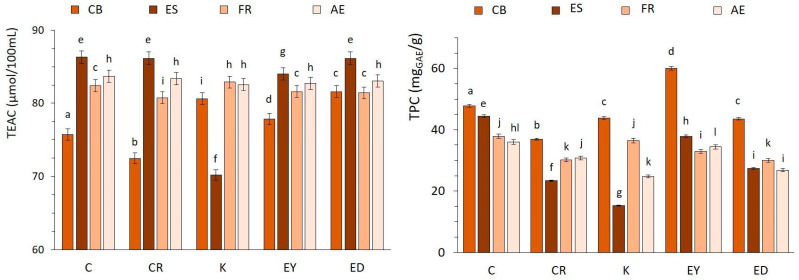
The radical scavenging activity of coffee extracts in reaction time of 60 min, expressed as Trolox equivalent antioxidant capacity (TEAC) (with error bars ≤ 1%), and total phenolic content (TPC) in coffee extracts, expressed in mg of gallic acid equivalent (GAE)/g of coffee (with error bars ≤ 1%). Different letters above the bars represent significant differences between the coffee samples (Tukey test, α ≤ 0.05).

The inactivation activity of coffee brews, as indicated by the DPPH % inhibition, was assessed over time at intervals of 0, 15, 30, 45, and 60 min. After 60 min (equilibrium time), most samples displayed radical scavenging activities within the range of 70–90%. These values were consistent with the coffee antioxidant activity levels reported by Kulapichitr et al. [39] and Seow et al. [40]. The results were also expressed as Trolox equivalent antioxidant capacity (TEAC) values (Figure 1), which were in line with data from the research by Sacchetti et al. [41]. Statistically significant differences in TEAC values among the various extraction methods and between the different geographical coffee types were identified at a significance level of α ≤ 0.05 using ANOVA. The antioxidant activity results were affected by the preparation conditions, i.e., water extraction temperature, extraction time, roasting degree, and other parameters, as also discussed by Kameya [42].

The highest antioxidant activity was observed in ES, particularly for the C omni-roasted coffee type, with a value of (86.3 ± 0.7) μmol/100 mL. However, espresso types C, CR, and ED exhibited similar antioxidant activities with no statistically significant differences (*p* > 0.05). In contrast to this, the K espresso type exhibited the lowest TEAC value at (70.2 ± 0.6) μmol/100 mL, which can be attributed to its lower content of polyphenols and chlorogenic acids, as discussed in Section 3.2 and Section 3.3. For FR and AE from light and medium coffee roasts, similar values of antioxidant activity were observed, with the C light-roasted type providing the highest TEAC at (82.4 ± 0.4) and (83.7 ± 0.5) μmol/100 mL, respectively. The observations align with the fact that the total antioxidant activity results from a balance between the degradation of phenolic compounds and the generation of Maillard reaction products, such as melanoidins with antioxidant activity, occurring during coffee preparation, as reported by other authors [21,43]. As also noted by Samsonowicz et al. [33], the radical scavenging activity may be attributed to the content of extracted polyphenols (TPC), as further discussed in Section 3.2. For CB, the antioxidant activity values of all coffee variants were lower compared to those of the other coffee brews, ranging from (72.5 ± 0.6) to (80.6 ± 0.5) μmol/100 mL, demonstrating that the cold temperature extraction can result in samples of lower antioxidant activity.

### 3.2. Total Polyphenols Content (TPC) Assay by Folin–Ciocalteu Method

In Figure 1, the total polyphenolic content (TPC) for five coffee types prepared by various methods is presented. The highest polyphenol content was observed in CB and ranged from (36.89 ± 0.25) to (60.06 ± 0.49) mg GAE/g, indicating that applying cold water for an extended time period is a more effective extraction type. For ES, the total polyphenol content ranged from (15.21 ± 0.12) to (44.41 ± 0.35) mg GAE/g; for FR, it ranged from (26.81 ± 0.13) to (37.89 ± 0.31) mg GAE/g; and for AE, it ranged from (24.74 ± 0.16) to (35.97 ± 0.29) mg GAE/g. ANOVA analysis revealed statistically significant differences between the TPC values of the various coffee extraction methods (*p* ≤ 0.05), although the differences in relation to the coffee’s geographical origin were not statistically significant (*p* > 0.05). The TPC results were in agreement with the TPC data determined for coffee extracts in the investigations by Le-Thi et al. [44] and Fărcaş et al. [45].

Among the CB samples, the CR coffee type showed lower TPC values, as well as the K coffee type between espressos. This could be related to the reduced antioxidant activity of these samples, as demonstrated in Figure 1. The C coffee type exhibited a high TPC, which could be attributed to omni-roasting process of C coffee with limited polyphenol degradation, as suggested by Bekedam et al. [46]. This was consistent with the fact that lower TPC values determined for other espressos can be ascribed to a higher roasting degree (Table 2), leading to increased polyphenol degradation.

As mentioned by Samsonowicz et al. [33], the presence of polyphenols may significantly impact the antioxidant activity of the brews. In this study, the TPC of cold brew was comparatively higher, even though the antioxidant activity was slightly lower due to the CB extraction conditions. This was also consistent with the observations presented by Derossi et al. [47], who noted a high variability between TPC and the antioxidant activity of coffee brews. The authors reported that there was no correlation between antioxidant activity and polyphenol content, which is also the case of our study. The TPC values for ES in the present study were consistent with the results by Derossi et al. [47], highlighting the significant effect of the preparation method on coffee attributes, in combination with the coffee powder’s grinding level.

### 3.3. Caffeine and Chlorogenic Acid Contents

**Figure 2:**
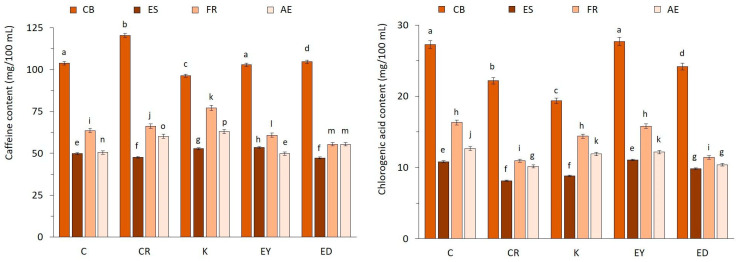
The caffeine and chlorogenic acid contents in coffee samples expressed in mg/100 mL (with error bars ≤ 1%). Significant differences are indicated by different letters above the bars (Tukey test, α ≤ 0.05).

The relatively large variations in caffeine and chlorogenic acid contents for the coffee samples under study can be attributed to the differences in brewing technique, roasting and grinding degree, coffee variety, and other factors [9,39,48]. ANOVA analysis revealed statistically significant differences (*p* ≤ 0.05) between the various extraction methods but insignificant differences between the coffee bean types (*p* > 0.05). The HPLC analysis was validated using the limits of detection (LOD) and quantification (LOQ). The LOD for caffeine was 0.2684 µg/mL, and the LOQ was 0.8134 µg/mL. For chlorogenic acid, the LOD was 1.010 µg/mL, and the LOQ was 3.062 µg/mL. For the HPLC data and a representative chromatogram related to the caffeine and chlorogenic acid contents, refer to Spreadsheet S1 in the Supplementary Materials. The cold brew (CB) method proved to be more effective in extracting caffeine in the final brew, with concentrations ranging from (96.3 ± 0.5) to (120.4 ± 0.9) mg/100 mL. For the other extraction methods, caffeine concentrations were substantially lower, regardless of the coffee type. Espresso (ES) yielded caffeine content in the range of (47.3 ± 0.3) to (53.5 ± 0.1) mg/100 mL, French press (FR) from (55.4 ± 0.1) to (77.0 ± 0.5) mg/100 mL, and aeropress (AE) from (49.8 ± 0.1) to (62.9 ± 0.2) mg/100 mL, as shown in Figure 2. These values correspond to the volume of coffee typically served in Central Europe. The caffeine content results can be compared with data by Parenti et al. [1] for espresso brews and Belitz et al. [48] for medium-roasted Arabica coffee.

For chlorogenic acid (CGA) content, the differences between the samples were statistically significant (*p* ≤ 0.05) regarding the various extraction methods and coffee bean types, as well (ANOVA). It was found that the CB extraction provided a relatively high CGA content, ranging from (19.4 ± 0.1) to (27.2 ± 0.2) mg/100 mL. The other extraction techniques yielded lower CGA values in the following order: ES from (8.12 ± 0.06) to (11.13 ± 0.10) mg/100 mL ˂ AE from (10.21 ± 0.09) to (12.70 ± 0.11) mg/100 mL ˂ FR from (10.91 ± 0.08) to (16.3 ± 0.15) mg/100 mL. These differences can be explained by the various conditions applied during the extraction procedures. The long maceration time (12 h) used in the CB technique allows for higher CGA extraction in the final brew compared to that of the shorter extraction times of ES, FR, and AE. These results, expressed in mg of CGA/g of coffee matter, were in agreement with published data on chlorogenic acid content from Klikarová et al. [9] and Belitz et al. [48] for roasted Arabica coffees. The filtered coffees (FR and AE) exhibited similar values of chlorogenic acid content, as shown in Figure 2.

### 3.4. Aroma Profile of Coffee Brews by HS-SPME/GC-MS

HS-SPME coupled with GC-MS was utilized to analyse the volatile compounds released from both the prepared coffee samples and the coffee powder (although the data for the coffee powder are not shown in this context). The analysis of the coffee powder was conducted to obtain reference spectra and retention parameters, primarily for comparative purposes, in an effort to enhance identification reliability. However, only data from the analysis of the prepared liquid coffee samples were used for factual evaluation and for the comparison of individual coffees and various coffee preparation methods (HS-SPME/GC-MS data of detected retention times and mass-to-charge ratios (*m*/*z*) are presented in Spreadsheet S2, Supplementary Materials). These data provided a more accurate characterization of the volatile substances responsible for the overall aroma of the prepared beverages, and they were better aligned with consumer perception. The main difference between the two types of samples lies in the lower extraction of water-soluble compounds from the liquid samples. This is due to their lower tendency to migrate to the headspace when compared to hydrophobic and less-water-soluble compounds [49].

A total of 98 substances were identified in the coffee aroma under these conditions, including twenty-five furans and furfurals, nineteen pyrazines, five pyrroles, two pyridines, three phenols, thirteen aldehydes, seventeen ketones, two acids, sixteen esters, five sulphur-containing compounds, and ten terpenes. This is consistent with the GC-MS results reported by Dong et al. [50], who classified the volatile compounds of roasted coffee beans into similar categories based on their chemical properties. The peak area corresponding to each compound was determined from an ion chromatogram reconstructed for the dominant ionic species specific to the particular analyte.

**Figure 3:**
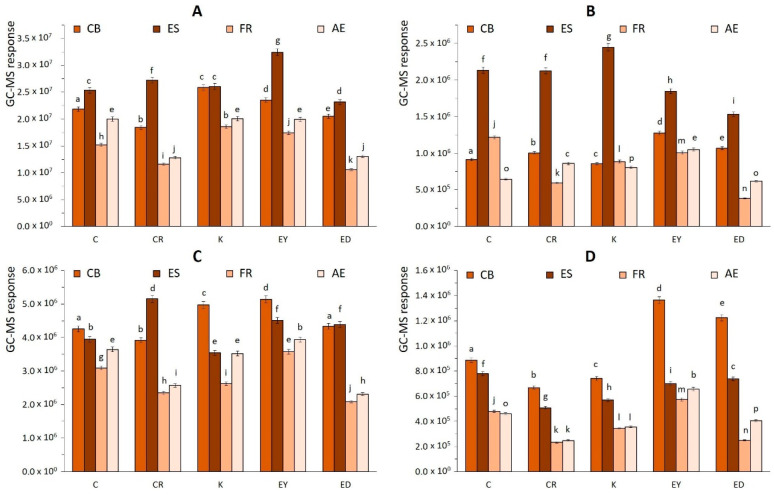
GC-MS responses of most represented coffee aroma compounds in coffee brews: (**A**)—furans, (**B**)—phenols, (**C**)—pyrazines, (**D**)—terpenes. Different letters above the bars (with error bars ≤ 2%) represent significant differences between the samples (Tukey test, α ≤ 0.05).

Figure 3 displays the sum of the peak areas of four groups of selected markers identified in coffee aromas. In Figure 3A, a group of six furan derivatives was selected, which represent typical products of the thermal decomposition of saccharides during roasting. This group included furfural, furfuryl alcohol, acetyl furan, 5-methyl-2-furfural, furfuryl acetate, and difurfuryl ether. The highest values were obtained for espresso samples, probably due to their having the highest extraction efficiency [51], and for cold brew, probably due to the low losses of volatile compounds during the extraction at the low temperature.

The second group (Figure 3B) included three methoxyphenols: guaiacol, 4-ethylguaiacol, and 4-vinylguaiacol. These compounds can be considered markers primarily derived from the thermal decomposition of lignin, chlorogenic acid, and related compounds. Similarly, the highest values were observed in espresso, followed by cold brew, with an exception for the C coffee type, where the second-highest content was observed in C French press. The more pronounced difference between espresso and cold brew can likely be attributed to the higher solubility of methoxyphenols in water at higher temperatures.

The third group (Figure 3C) comprised seven pyrazine compounds: pyrazine, 2-methylpyrazine, 2,6-dimethylpyrazine, 2,5-dimethylpyrazine, 2-ethyl-5-methylpyrazine, 2-ethyl-6-methylpyrazine, and 3-ethyl-2,5-dimethylpyrazine. These compounds may be clearly related to the pyrolysis of amino acids, peptides, and other amino acid derivatives. ES and CB showed higher values compared to those of FR and AE, with differences between individual coffee types. Especially in this pyrazine group, the observed phenomenon may be partially attributed to the behaviour of individual compounds. Compounds of low molecular weight that were more volatile and probably more soluble were well-extracted by both methods (ES and CB), but the losses caused by volatilization may be higher during the long extraction time, even at a lower temperature (pyrazine, 2-methylpyrazine) [52]. Compounds of higher molecular weight that were less volatile and probably less soluble required a longer time for quantitative extraction and provided higher peaks after long extraction times even at the lower temperature during the cold brew preparation (2-ethyl-6-methylpyrazine and 3-ethyl-2,5-dimethylpyrazine).

The same phenomenon was observed to some extent with other substances from different groups and was most evident in the last group of terpenic compounds (Figure 3D), which included linalool oxide, linalool, and geraniol. These compounds are considered markers primarily associated with coffee beans themselves and the biological or thermal decomposition of some lipids. All three selected terpenes were less volatile than the selected pyrazines, and the highest values of the sums of peak areas were consistently obtained for cold brew.

When considering the sum of the peak areas of all selected markers across all types of coffee and extraction methods, the EY and K provided the highest content of volatile organic compounds, followed by C, ED, and CR. Coffee type EY, characterized by medium-dark roasting and finer grinding of espresso coffees, may be considered optimal for releasing higher levels of aroma compounds, as detected by GC-MS.

**Figure 4:**
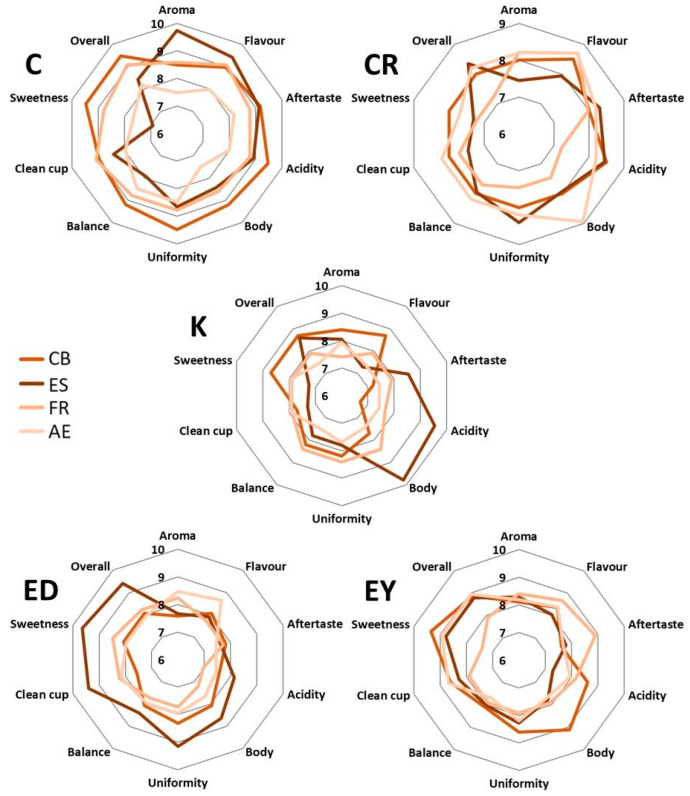
Sensory attribute evaluations of coffee samples based on coffee type (geographical origin) and coffee brew preparation, as indicated in the legend. The numbers in radar chart subsections represent the average scores of sensory attributes according to the Specialty Coffee Association (SCA) standard protocol.

Some organic acids (acetic and isovaleric acid), sulphides, and terpenoids were detected, as well. In terms of the coffee brew preparation methods, the extraction effectiveness of the volatile compounds was as follows: ES > CB > AE > FR. This can be related to the higher effectiveness of the ES and CB methods in extracting antioxidant substances, total polyphenolic content, caffeine, and chlorogenic acid. It aligns with the findings of De Vivo et al. [53], who noted that espresso volatile compounds are released differently depending on the coffee species and particle size, similar to the present study. The results of the coffee aroma profiles were correlated with sensory analysis data, as presented in Figure 4. Since coffee’s volatile compounds significantly influence the in-cup profile [51], the levels and composition of GC-MS fractions were closely related to the coffee sensory properties and aroma perception [10,49]. The ES and CB samples, which had more intense aroma profiles, were preferred over filtered coffees (FR and AE), as discussed in Section 3.5.

### 3.5. Sensory Evaluation of Coffee Brews

Sensory testing was conducted to characterize the differences between the coffee brews. The samples were rated on a numerical scale according to SCA classification, as described in Section 2.10. Coffee brews which received higher scores were evaluated as samples of significantly higher quality compared to coffees with lower scores [36]. The results, rated as total scores by the members of the sensory panel, showed that only the C cold brew could be classified as outstanding coffee with an average total score of (91.86 ± 1.82). The other C coffees were rated as excellent (ES, FR) or very good (AE). Compared to the other coffee types, no C sample was below specialty quality, which could be attributed to the omni-roasting process of C coffee beans; this roasting seemed to provide the most desirable sensory properties to various coffee brews. ED was assessed as the least preferable coffee type with the samples being below specialty coffee quality, except espresso. In general, ES and CB were classified as more desirable methods and FR and AE as less preferable, producing some samples below specialty coffee quality (ED French press and aeropress, CR French press, and K aeropress); the lowest average total scores were assigned to K aeropress (76.00 ± 2.22) and CR French press (76.82 ± 1.64). The results of our study were consistent with the findings of Bhumiratana et al. [23] who confirmed that the sensory aroma profiles of coffee brews were more influenced by the preparation stages, including the roasting and grinding degree, than the coffee variety.

The individual sensory attributes of the coffee samples were compared as average scores (in the range 6–10), as illustrated in Figure 4. From an aroma point of view, C omni-roasted coffee was preferred as the most favourable to prepare all coffee brew types, except aeropress. The aroma of C espresso was rated as the most desirable sensory attribute (9.73 ± 0.10) under study. In the case of the K coffee type, the differences in aroma preferences between the brew types were only minor (8.25 ± 0.11). In contrast to this, for the ED and CR coffee types, FR and AE were rated as more favourable preparations compared to CB and ES.

Despite the highly rated sensory profiles of the C omni-roasting type, the coffee type C was rated as the least acceptable espresso in terms of sweetness, especially when compared to ES prepared from medium-dark roasts such as CR, EY, and ED. This observation is in line with the idea that certain attributes can have reduced acceptability when the coffee is roasted at lower temperatures, as suggested by Laukaleja and Kruma [43]. The dark-medium-roasted espressos, CR, EY, and ED, generally provided higher scores for sweetness compared to espressos roasted at lower temperatures like C and K espressos. This observation can be related to the bitter sensory characteristics of melanoidins and chlorogenic acids lactones mostly formed at lower roasting levels, reducing the coffee cup quality [43]. Surprisingly, despite the influence of roasting temperature, the acidity and body of K espresso (light-medium roast) and C omni-roasts (except C aeropress) received higher ratings compared to the other coffee samples. This suggests that coffee acidity can be well-accepted in conjunction with bitterness, highlighting acidity as an essential sensory attribute [24].

In conclusion, the most preferred coffee sample among all those studied was C cold brew, which received the highest average ratings for sensory attributes, specifically for acidity, uniformity, sweetness, and overall, with a combined score of (9.48 ± 0.15). Upon statistical evaluation by the Pearson test, the sensory analysis data demonstrated a significant, dependent relationship between the variables (*p* ≤ 0.05). With a 95% confidence level, it was established that the coffee type and preparation method had a statistically significant impact on the sensory profile attributes, as assessed by the sensory panel members.

## 4. Conclusions

The present study underscores the critical importance of coffee extraction methods in determining the final composition of coffee brews, which, in turn, significantly influences the brews’ antioxidant activity and aroma profile. The complex aroma profiles of the coffee brews were composed of various volatile compounds, with the most abundant ones being furfural, 5-methyl-2-furfural, methylpyrazine, 2,5-dimethylpyrazin, and 1-methyl-1H-pyrrole-2-carboxaldehyde. Among the alcohol and phenol substances, furfuryl alcohol and 2-methoxyphenol were the predominant compounds, respectively. Additionally, some organic acids, sulphides, and terpenoids were detected.

When comparing extraction methods, the espresso (ES) and cold brew (CB) extractions consistently yielded higher levels of volatile compounds than French press (FR) and aeropress (AE). Espresso, particularly the EY medium-dark roasting type, exhibited the highest levels of most coffee volatiles. Members of the sensory panel preferred ES and CB as more desirable coffee extraction methods for the majority of the coffee variants. Notably, the Colombia cold brew coffee was rated as an outstanding quality sample. In contrast, some of the filtered coffees, such as ED (French press and aeropress), CR (French press), and K (aeropress), were classified as falling below the standard of specialty coffee quality. The geographical origin of the coffee also played a role in preferences, with the Colombia omni-roasted type being the most favoured sample. ES and CB were found to be more effective extraction methods, particularly with regard to their total polyphenolic content (TPC), caffeine content, and chlorogenic acid content. ES and CB also showed higher values of antioxidants compared to FR and AE. The Colombian ES type (omni-roast) exhibited the highest antioxidant activity (86.31 ± 0.70 mmol/100 mL), and its TPC value was (44.41 ± 0.35 mg GAE/g).

In conclusion, this research provides insights into the role of extraction methods in influencing the aroma profile, antioxidant activity, and overall quality of coffee brews. The work elucidates the significant role of extraction methods in shaping the sensory characteristics of the brews. The findings, therefore, offer implications for enhancing coffee brews’ aroma profile and sensory quality in view of the extraction process applied.

## Figures and Tables

**Table 1:** Characterization of coffee bean geographical origins (as provided by the producers).

Coffee Beans Geographical Origin					
	Columbia (C)	Costa Rica (CR)	Kenya (K)	Ethiopia Yirgacheffe (EY)	Ethiopia Daro Kebele (ED)
Variety	Castillo	Catuaí	SL28	Kurume	Kudhume, Welisho, Dego
Cultivation area	Quindio	Tarrazu	Nyeri	Yirgacheffe	Daro Kebele
Cultivation altitude (m.a.s.l.)	1450	2200	1880–1970	2000–2050	1700–2200
Processing type	Carbonic maceration	White honey processing	Wet processing (washing)	Wet processing (washing)	Dry (natural) processing
Flavour profile	Papaya, strawberry, orange peel	Peach, red apple, caramel	Black currant, hibiscus, orange	Chamomile, bergamot, Earl Grey tea	Violet, strawberry, vanilla
Producer	Finca Peurto Alegre	La Pastora, Carlos Montero	Thiriku Cooperative	Tessema Edima	Abado

**Table 2:** Characterization of coffee bean grinds and roasts in relation to coffee brewing methods.

Grinding Degree		Roasting Degree of Coffee Beans				
Coffee Brew Type (Abbrev.)	${\bar{r_{w}}}^{a}$ (μm)	C	CR	K	EY	ED
Cold brew (CB)	215	Omni-roast (light)	Medium	Medium	Medium	Medium
Espresso (ES)	185	Omni-roast (light)	Medium dark	Medium light	Medium dark	Medium dark
French press (FR)	204	Omni-roast (light)	Medium	Medium	Medium	Medium
Aeropress (AE)	208	Omni-roast (light)	Medium	Medium	Medium	Medium

${\bar{r_{w}}}^{a}$—weight average (mean) particle radius (grind size).

## Data Availability

The HPLC and HS-SPME/GC-MS datasets generated in this study are available in Supplementary Materials.

## Supplementary Materials

The following supporting information can be downloaded at: https://www.mdpi.com/article/10.3390/foods12224125/s1, Spreadsheet S1: HPLC data and a representative chromatogram of caffeine and chlorogenic acid contents in coffee brews samples; Spreadsheet S2: HS-SPME/GC-MS data related to the volatile compounds detected in coffee brews samples.
